# Anatomy, morphology and evolution of the patella in squamate lizards and tuatara (*Sphenodon punctatus*)

**DOI:** 10.1111/joa.12435

**Published:** 2016-01-06

**Authors:** Sophie Regnault, Marc E. H. Jones, Andrew A. Pitsillides, John R. Hutchinson

**Affiliations:** ^1^ Department of Comparative Biomedical SciencesThe Royal Veterinary CollegeLondonUK; ^2^ School of Biological SciencesThe University of AdelaideAdelaideSAAustralia; ^3^ South Australian MuseumAdelaideSAAustralia

**Keywords:** ancestral state reconstruction, bone, histology, Lepidosauria, ossification, palaeontology, Rhynchocephalia, sesamoid

## Abstract

The patella (kneecap) is the largest and best‐known of the sesamoid bones, postulated to confer biomechanical advantages including increasing joint leverage and reinforcing the tendon against compression. It has evolved several times independently in amniotes, but despite apparently widespread occurrence in lizards, the patella remains poorly characterised in this group and is, as yet, completely undescribed in their nearest extant relative *Sphenodon* (Rhynchocephalia). Through radiography, osteological and fossil studies we examined patellar presence in diverse lizard and lepidosauromorph taxa, and using computed tomography, dissection and histology we investigated in greater depth the anatomy and morphology of the patella in 16 lizard species and 19 *Sphenodon* specimens. We have found the first unambiguous evidence of a mineralised patella in *Sphenodon*, which appears similar to the patella of lizards and shares several gross and microscopic anatomical features. Although there may be a common mature morphology, the squamate patella exhibits a great deal of variability in development (whether from a cartilage anlage or not, and in the number of mineralised centres) and composition (bone, mineralised cartilage or fibrotendinous tissue). Unlike in mammals and birds, the patella in certain lizards and *Sphenodon* appears to be a polymorphic trait. We have also explored the evolution of the patella through ancestral state reconstruction, finding that the patella is ancestral for lizards and possibly Lepidosauria as a whole. Clear evidence of the patella in rhynchocephalian or stem lepidosaurian fossil taxa would clarify the evolutionary origin(s) of the patella, but due to the small size of this bone and the opportunity for degradation or loss we could not definitively conclude presence or absence in the fossils examined. The pattern of evolution in lepidosaurs is unclear but our data suggest that the emergence of this sesamoid may be related to the evolution of secondary ossification centres and/or changes in knee joint conformation, where enhancement of extensor muscle leverage would be more beneficial.

## Introduction

The tibial patella (‘kneecap’: hereafter referred to simply as the patella) is a sesamoid; a bone found within a tendon or ligament as it passes around a joint. Sesamoids are hypothesised to confer biomechanical advantages, such as increasing a muscle's moment arm, as well as protecting tendons as they wrap around joints (Sarin et al. 1999). The patella is generally the largest and most familiar sesamoid bone, and has evolved on at least three independent occasions: in mammals, birds and squamate lizards (Haines, 1940; Dye, 1987). Lizards (and some birds and mammals) also possess an ‘ulnar patella’ in the forelimb: a sesamoid positioned in the elbow similarly to the tibial patella of the knee (both found within the tendon of the principal extensor muscle for their respective joints).

Early studies were mainly concerned with the largest lizard sesamoids, and claimed that the patella was only occasionally present (Cope, 1892; Parsons, 1908; de Vriese, 1909; Pearson & Davin, 1921; Camp, 1923; Romer, 1956, 1997). Contrastingly, Maisano (2002a), Jerez et al. (2010), and Otero & Hoyos (2013) explicitly aimed to clarify and compare the anatomy and evolution of many sesamoids in lizard taxa, and reported widespread distribution of the patella in Squamata. Fossilized patellae have been reported in squamate reptiles such as mosasaurs and their relatives (e.g. *Carsosaurus* by Kornhuber, 1893), the iguanian *Saichangurvel* (Conrad & Norell, 2007), and anguimorphans (e.g. *Saniwa* by Rieppel & Grande, 2007; *Dalinghosaurus* by Evans et al. 2007; *Gobiderma* by Conrad et al. 2011), so a patella dates back at least to the Mesozoic era. But several key questions remain. What exactly is the structure and composition of the squamate patella? When did it first evolve? Which species possess it and how did the patella evolve within lizards or closely related diapsid reptiles (e.g. early lepidosauromorphs)? Finally, under what circumstances did the patella evolve, and why might it have been lost or never evolved in other sprawling reptiles where it might offer similar biomechanical advantages (e.g. turtles, crocodiles)? Here, we aim to begin addressing these questions as much as evidence allows, through imaging and histology of extant taxa and evaluation of osteological and fossil specimens. Although we cannot answer the latter question with distribution data alone, it is the first step in establishing an evolutionary hypothesis.

Few studies have investigated the patellar structure in lizards, beyond noting its presence or absence. These studies observed that the squamate patella is usually bony (Haines, 1942a, 1969) and may form via endochondral ossification of a cartilaginous precursor, similar to the patellae of mammals and birds. Further in‐depth examination of the patella in lizards is warranted, however, to test this assertion, because the composition (bone, mineralised tendon, fibrous tissue or cartilage) and development of the patella in lizards is unclear (Otero & Hoyos, 2013). Additionally, the possibility of a soft tissue analogue in lizards or other reptiles lacking bony patellae (such as the patelloid in marsupials; Reese et al. 2001) has not been explored by any study, aside from brief mentions of a ‘patelloid’ mass in a crocodylian (Parsons, 1908) and turtle (*Terrapene carolina*; Haines, 1969). An interesting related question is whether the ossified patella might have evolved from a precursor (such as tendon fibrocartilage or a patelloid‐like structure); evidence on this issue likewise remains lacking.

The patella is hypothesised to be ancestral for squamates (Maisano, 2002a; Vickaryous & Olson, 2007), but the possibility of patellae in their sister group, Rhynchocephalia, has not been systematically investigated. Rhynchocephalia was once a diverse and globally distributed clade (e.g. Jones, 2008; Jones et al. 2009), but today it is represented only by a single living species: the tuatara (*Sphenodon punctatus*) found in New Zealand (Hay et al. 2010). These lizard‐like reptiles are a valuable reference taxon for exploring the evolution of difficult‐to‐preserve traits, such as the patella, in squamates and other groups (Maisano, 2002a; Jones & Cree, 2012). Older literature is essentially unanimous that *Sphenodon* lacks a patella; in detailed anatomical descriptions, such a structure is either not included in text and figures (Perrin, 1895; Howes & Swinnerton, 1901; Haines, 1942a) or explicitly noted as absent (Günther, 1867; Osawa, 1897; von Wettstein, 1931). Parsons (1908) noted chondrification of the patellar tendon in tuatara, but no mineralisation (i.e. calcification or ossification). The patella is ‘unknown’ in *Sphenodon* in Gauthier et al.'s (2012) extensive character matrix, along with many other sesamoids. No modern studies have explicitly tested the claim that *Sphenodon* lacks a patella. Also, given that extinct members of Rhynchocephalia exhibit variation in body shapes that likely reflect different lifestyles (Reynoso, 2000) the character state in *Sphenodon* cannot be extrapolated to all Rhynchocephalia. However, should patellae be discovered in *Sphenodon* or its relatives, there is the potential for an earlier evolutionary origin of the patella in the common ancestor of squamates and rhynchocephalians (together constituting the clade Lepidosauria).

There is reason to expect a patella in *Sphenodon*. Like the patella, secondary epiphyseal ossification centres have also evolved on repeated occasions, and generally appear to co‐occur with sesamoids in many groups (Sarin et al. 1999), suggesting that these two features are linked by some yet obscure developmental mechanism (Carter et al. 1998). Non‐avian dinosaurs, crocodiles, turtles and salamanders lack ossified epiphyses and sesamoids (though they may have cartilaginous sesamoid structures, e.g. Tsai & Holliday, 2011), whereas lizards, mammals, anurans and birds (variably in the latter cases) tend to possess both ossified epiphyses and sesamoids (Haines, 1938, 1942b; Carter et al. 1998; Ponssa et al. 2010). Terrestrial Rhynchocephalia such as *Sphenodon* also possess epiphyseal ossifications (Moodie, 1908; Gauthier et al. 1988) and several smaller sesamoids have been noted in *Sphenodon* (e.g. on the dorsal surfaces of the penultimate phalanges; characters 547 and 569 in Gauthier et al. 2012).

The purported link between secondary centres of ossification and sesamoids (Carter et al. 1998) may hold clues, via the underpinning mechanism(s) regulating both, as to why lizards possess patellae and other animals do not. Another explanation may be provided by the phylogenetic differences in locomotor style or posture, in which a novel mechanical environment favours patellar formation (due to tissue metaplasia from structures experiencing differing forces (e.g. Giori et al. 1993; Benjamin et al. 1995; Sarin et al. 1999) or in which a patella would be favourable (by conferring biomechanical advantages that were previously not beneficial or otherwise absent).

In this study, we combine multiple lines of evidence to better characterise patellar evolution in Lepidosauromorpha and begin answering some of the outstanding questions regarding patellar origins in these species. We use both long established and advanced imaging methods to document patellar presence and morphology in tuatara and diverse lizard species, and histology to investigate patellar composition. We consolidate existing datasets of patellar presence in squamates and use these with our own observations to reconstruct likely ancestral character states and estimate the patella's evolutionary origin. We evaluate the patterns of loss and gain within Lepidosauria, and hypothesise why the patella may have evolved in this clade and not others.

## Materials and methods

Nineteen alcohol‐preserved tuatara (*Sphenodon* sp.) with intact hindlimbs underwent X‐ray microtomography scanning (XMT) at the University Museum of Zoology Cambridge (UMZC), using an XT H 225 ST computed tomography system (Nikon Metrology, Brighton, MI, USA). The patellar tendon was sampled from three of these specimens (specimen ‘S1’, specimen ‘S15’ and BMNH1969.2204); one specimen (‘S1’) with evidence of patellar mineralisation underwent further high‐resolution XMT scanning at the Royal Veterinary College (RVC) using a Skyscan 1172 (Bruker microCT, Kontich, Belgium).

The leg and/or patellar tendon from 16 lizard specimens, belonging to University College London (UCL) and RVC, representing a variety of squamate clades also underwent high‐resolution XMT scanning at the RVC. One large specimen (*Varanus komodoensis*) underwent computed tomography (CT) scanning at the RVC using a Lightspeed Pro 16 machine (GE Medical, UK). Specimen details and scanning parameters are listed in Supporting Information Table S1. In addition to noting patellar presence and morphology, specimens were examined for terminal fusion of the long bone epiphyses. In squamates, the latter is an indication that maximal size and cessation of growth is near or has been achieved (though exceptions exist) (Maisano, 2002b) and can be used to judge the skeletal maturity of an individual. The apparently late formation of the patella relative to other bones (Maisano, 2002a; Jerez et al. 2010), led us only to count presence/absence of the patella in specimens with complete, near‐complete and incomplete terminal fusion, and discount patellar absence in the small number of specimens with very early epiphyseal ossification (though these are still detailed in Supporting Information Data S1). Likewise we excluded specimens with early epiphyseal ossification from the ancestral state reconstruction, described below.

The patellar tendons of the above scanned squamates and three individual *Sphenodon* specimens were prepared for histological examination. Those tendons exhibiting mineralisation (evident from the X‐ray CT images) were decalcified in a 14% EDTA solution for 1 week, with the endpoint confirmed by XMT scanning. Specimens were embedded in wax and serially sectioned. The sections were then stained with standard Haematoxylin and eosin (H&E) and Safranin O/Fast green.

Whole preserved lizards and tuatara belonging to NHM London and the University of Adelaide were radiographed with various settings for optimal bone visualisation (generally 30–32 kV and 11–18 s). Osteological specimens belonging to NHM London were also examined and photographed. Specimen numbers are detailed in Supporting Information Data S1.

Collections of fossil Rhynchocephalia and stem Lepidosauria (non‐lepidosaur lepidosauromorphs) and other Reptilia belonging to Museum für Naturkunde (MfN) Berlin, Staatliches Museum für Naturkunde (SMNS) Stuttgart, Natural History Museum London (NHMUK), and UMZC were examined for evidence of patellar mineralisation. Specimens are listed in Table S2.

For ancestral state reconstruction, we coded lepidosauromorph taxa based on our own collected data and the literature; publications detailing patellar presence in squamate species were a rich source of data in building the character matrix (Camp, 1923; Haines, 1942a; Mohamed, 1988; Maisano, 2002a; Conrad, 2006, 2008; Jerez & Tarazona, 2009; Jerez et al. 2010; Daza et al. 2012; Gauthier et al. 2012; Otero & Hoyos, 2013). Patellar character states were coded such that ‘0’ = ossified patella absent, ‘1’ = ossified patella present, ‘0/1’ = polymorphic (variable within/between individuals), ‘?’ = unknown patellar state, ‘–’ = not applicable (due to reduced or absent hindlimbs; e.g. snakes, dibamids). Where conflicts existed between the published and observed data, we coded the patella as it appeared in our data, or as polymorphic (‘0/1’) or uncertain (‘?’). Parsimony reconstruction was performed over a composite tree built manually from the recent phylogenies of Reeder et al. (2015) and Pyron et al. (2013) in mesquite software (Maddison & Maddison, 2015). We also explored the sensitivity of our reconstructions to tree topology using an alternative morphology‐based phylogeny (Gauthier et al. 2012) and to character state coding by observing the changes in trait evolutionary history when different character coding was used for ambiguous or polymorphic taxa.

A note on anatomy: in general, the patellar tendon (continuous with the triceps tendon) in lepidosaurs is formed by contributions from the thigh muscles *M. femorotibialis externus* and *M. ambiens*, with smaller contributions from *M. femorotibialis internus*,* M. iliotibialis* and fascia connecting to the lower limb muscles (S. Regnault & J. R. Hutchinson, pers. obs., 2015). This is similar to the state observed in birds (Regnault et al. 2014) but with the increased role of *M. ambiens* (relative to the predominance of the lateral head of the femorotibial muscle in birds) and the weaker connection of lower limb muscles. We have observed grossly similar connections in Crocodylia (with the triceps tendon only, e.g. Allen et al. 2014), so these connections in lepidosaurs may be plesiomorphic for the broader clade Sauropsida.

## Results

### The patella in *Sphenodon* (Rhynchocephalia)

Four of the 19 XMT‐scanned tuatara in this study were found to possess a discrete region of patellar mineralisation in both hindlimbs. It was not clear whether the mineralised regions comprised calcifications or ossifications, so we have used the term ‘mineralisation’ where this was the case. These apparent patellae were located superficially over the cranial (dorsal) distal femur in the patellar tendon, which matches the position of the patella in lizards (see below). All four individuals had complete (or near‐complete) fusion of their femoral epiphyses as judged from XMT scans. Of the remaining 15 individuals found to lack any clear mineralisation in the patellar tendon, seven had complete terminal epiphyseal fusion and eight did not. A tuatara from the University of Adelaide teaching collection was also radiographed, but unlike the CT‐scanned specimens there was no clear evidence of mineralised patellae.

**Figure 1:**
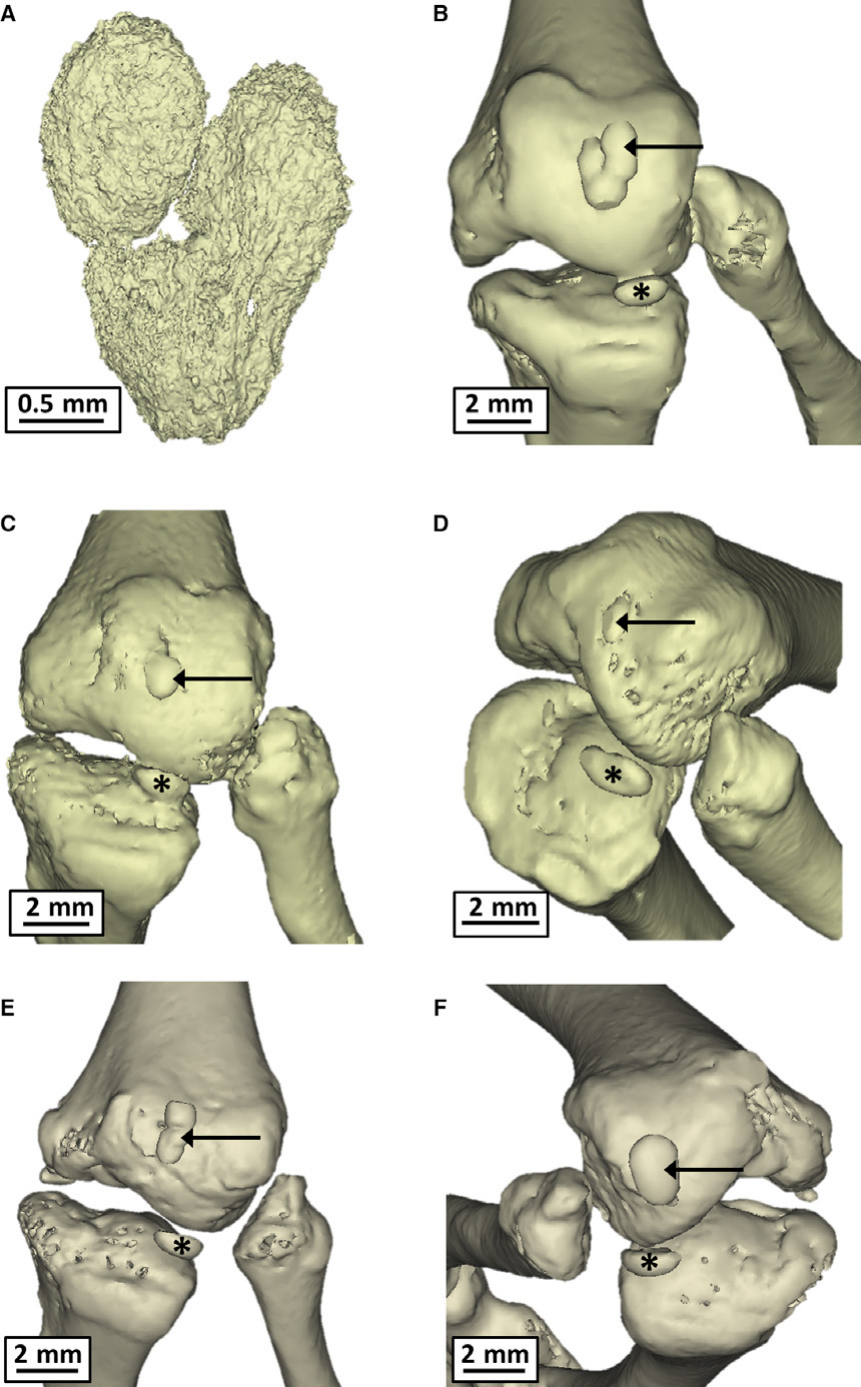
3D reconstructed models of the XMT‐scanned patellar mineralisations in *Sphenodon punctatus* (arrow pointing at patella; specimen details in Table 1). (A) High‐resolution XMT of the right patella in specimen ‘S1’. (B) Left patella scanned *in situ* from ‘S1’. (C) Left patella *in situ* from specimen R.2604. (D) Left patella *in situ* from specimen BMNH1935.12.6.1. (E,F) left and right patellae *in situ* from specimen NH.84.19. Also visible in these specimens is a tibial lunula (asterisk).

Morphology of these patellar mineralised regions was variable (Fig. 1). In specimen ‘S1’, both patellar mineralisations were tri‐lobed and symmetrical between limbs (Fig. 1A,B). In two of the tuatara (R.2604 and BMNH1935.12.6.1), the mineralised region had a flattened ovoid shape in both limbs (Fig. 1C,D). In NH.84.19, the right patellar mineralisation had a similar ovoid morphology (Fig. 1F) but the left was proximo‐distally bi‐lobed (Fig. 1E). The dimensions of the patellar mineralisations are shown in Table 1. There is no obvious correlation between patellar length and femur length, albeit our data are limited.

**Table 1:** Summary of tuatara (*Sphenodon*) specimens with patellar mineralisations

Specimen	Femur length (mm)	Left patellar description (measurements: height × width in mm)
R.2604 (UMZC)	32.1	One mineralisation (1.3 × 1.0)
NH.84.19 (HM)	35.2	Proximodistally bi‐partite but fused mineralisation (2.1 × 0.8)
‘S1’ (MEHJ personal collection ID; UCL)	37.6	Tri‐partite fused mineralisation (2.7 × 1.8)
BMNH1935.12.6.1 (NHM)	43.4	One mineralisation (1.1 × 0.5)

**Figure 2:**
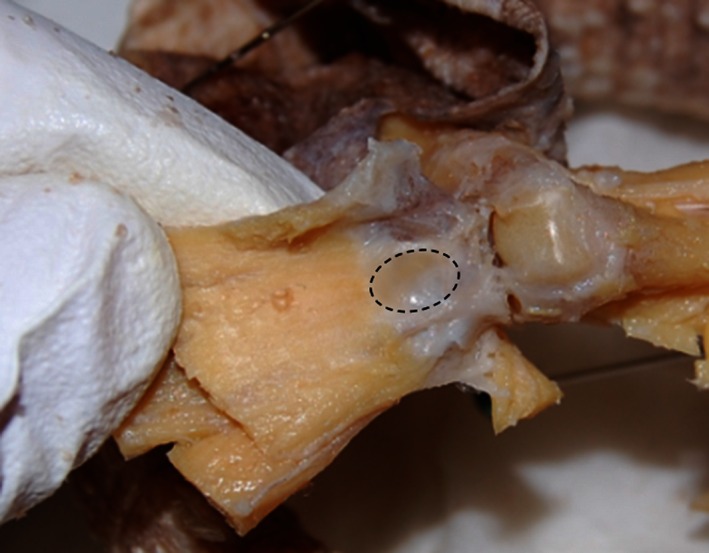
Gross appearance of the patellar tendon reflected from the distal femur in *Sphenodon punctatus* specimen ‘S1’, and in which the concave deep surface of the patellar mineralisation (marked by dotted outline) is subtle yet appreciable. Not to scale.

**Figure 3:**
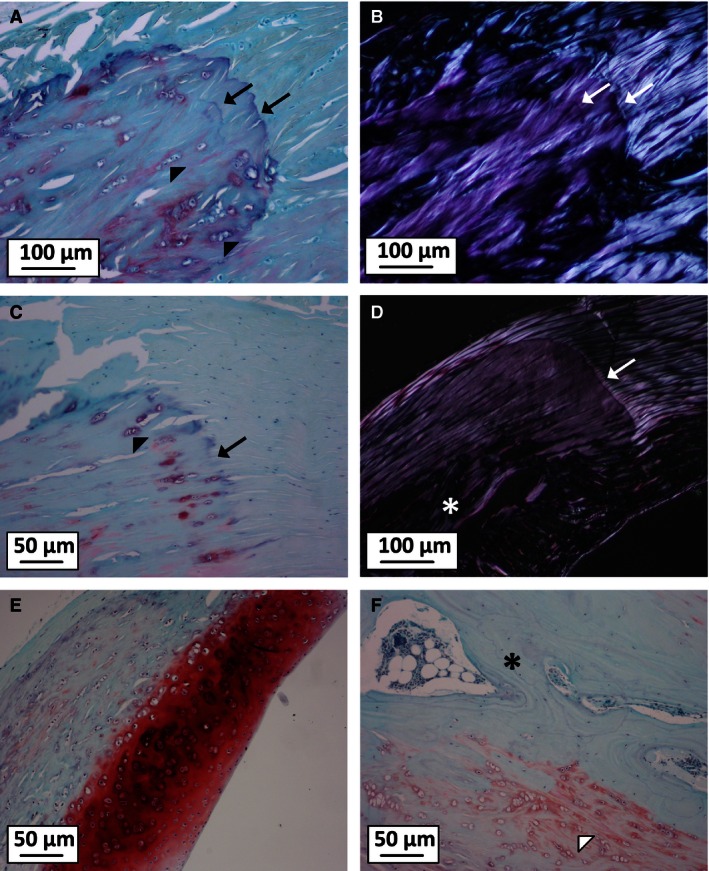
Histological appearance of the patellae in Lepidosauria, stained with Safranin O/Fast green. (A) *Sphenodon* specimen ‘S1’, showing the mineralised border (as well as faint previous tidemarks; arrows) with continuous tendon fibres crossing it and rows of chondrocyte‐like cells (arrowhead). (B) The same view of *Sphenodon* specimen ‘S1’ under polarised light, highlighting the tendon fibres which cross the tidemarks (white arrows). (C) *Hydrosaurus pustulatus* research ID ZR/922/10, which has a similar composition to the patella of *Sphenodon* specimen ‘S1’. (D) *Tiliqua scincoides* (no research ID) under polarised light, showing continuous tendon fibres across the tidemark (white arrow). The deep part of the patella in this specimen is formed of bone (asterisk), and the collagen fibres here can be seen to be discontinuous with those of the tendon. (E) *Gekko gecko* (no research ID), which appears as a mass of calcified hyaline cartilage. (F) *Corucia zebrata* research ID ZR/935/10 shows both lamellar bone (blue, asterisk) and calcified tendon with cartilaginous changes (pink, unfilled arrowhead).

The patellar tendon was removed from three tuatara: specimen ‘S1’ (in which the mineralisation was appreciable; Fig. 2, confirming that it was indeed within the patellar tendon), and specimens ‘S15’ and BMNH1969.2204 (two individuals without mineralisation). In the two specimens without patellar mineralisation, serial histological sections showed no evidence of a patella anlage or precursor; the patellar tendon appeared to consist of conventional, dense parallel collagen fibre bundles with few cells and without signs of cartilage or bone formation. In specimen ‘S1’, histological evidence for mineralisation was found based upon a basophilic ‘tidemark’ that coincided spatially with the demarcation of the patella border (Fig. 3A). Tendon fibres appeared continuous across the tidemark in this specimen (Fig. 3B), with small clusters and columns of chondrocyte (or chondrocyte‐like) cells in lacunae around and within the patellar mineralisation. A similar appearance is seen in some squamates, though not all (Fig. 3C‐F; see also below).

For ancestral state reconstruction, *Sphenodon* was initially assigned a patellar state of ‘0/1’ in mesquite (mineralised patella absent and present) to reflect its apparent polymorphism. However, alternate codings (i.e. 0 or 1) were also fully explored in sensitivity analysis and had significant consequences for ancestral reconstructions of patellar state in Lepidosauria – see below and Discussion.

### The patella in Squamata

**Figure 4:**
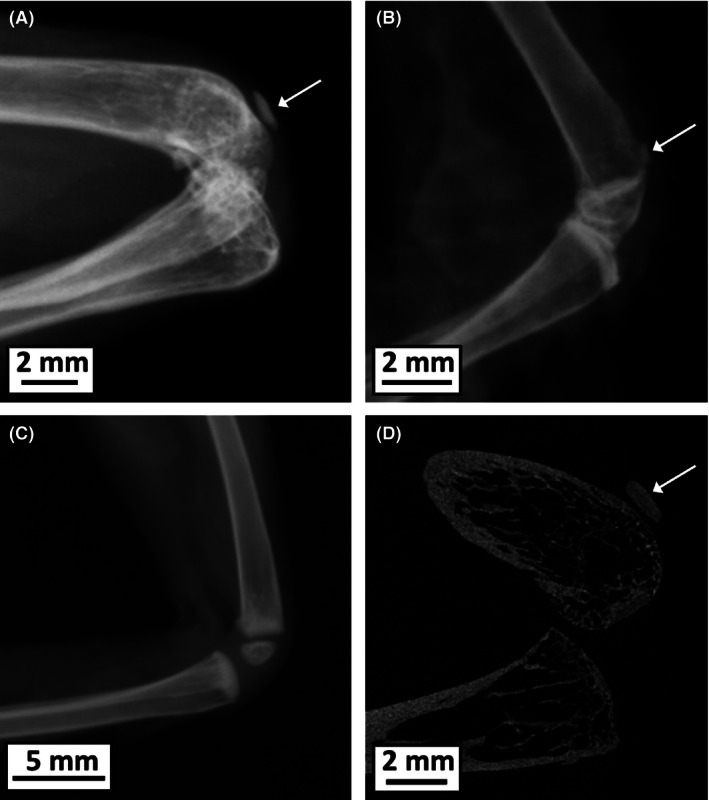
Most lizards with patellae had complete (no visible separate epiphysis; e.g. radiograph of *Brachylophus subcristatus* South Australian Museum (SAMA) number 66002, (A)) or near complete (physis visible but almost fully fused; e.g. radiograph of *Sceloporus jarrovii* SAMA number 66681, (B)) terminal epiphyseal fusion. Lizard specimens with early epiphyseal ossification (rounded ossification centres with large gaps between the diaphysis; e.g. radiograph of *Basiliscus plumifrons* SAMA number 40103, (C)) did not show evidence of mineralised patellae. Another, more terminally fused *Basiliscus plumifrons* specimen with patellae [e.g. XMT slice of *B. plumifrons* research ID ZR/519/09 (D)] supports the idea that this sesamoid mineralises later in ontogeny. Arrows show patellae.

From literature data, museum studies, and specimen imaging, we found the majority of squamates studied to possess a mineralised patella; see Supporting Information Data S1 for our patellar character state data for each species, with sources for those data. Lizards with patellae generally had complete or near‐complete terminal epiphyseal fusion (where the epiphysis was not visible or very nearly fused; Fig. 4A,B); 46 lizards (from 45 species) with clear patellae had fused or near‐fused epiphyses (~ 70% of the 66 individuals/63 species sampled). Fewer lizards had a mineralised patella with incomplete epiphyseal fusion; two lizards (two species). No lizards had evidence of patellae with early epiphyseal ossification (where the epiphyseal ossification centre was rounded with a large gap between it and the long bone shaft; Fig. 4C), although the patella sometimes appeared in more mature specimens of the same species (Fig. 4D).

**Figure 5:**
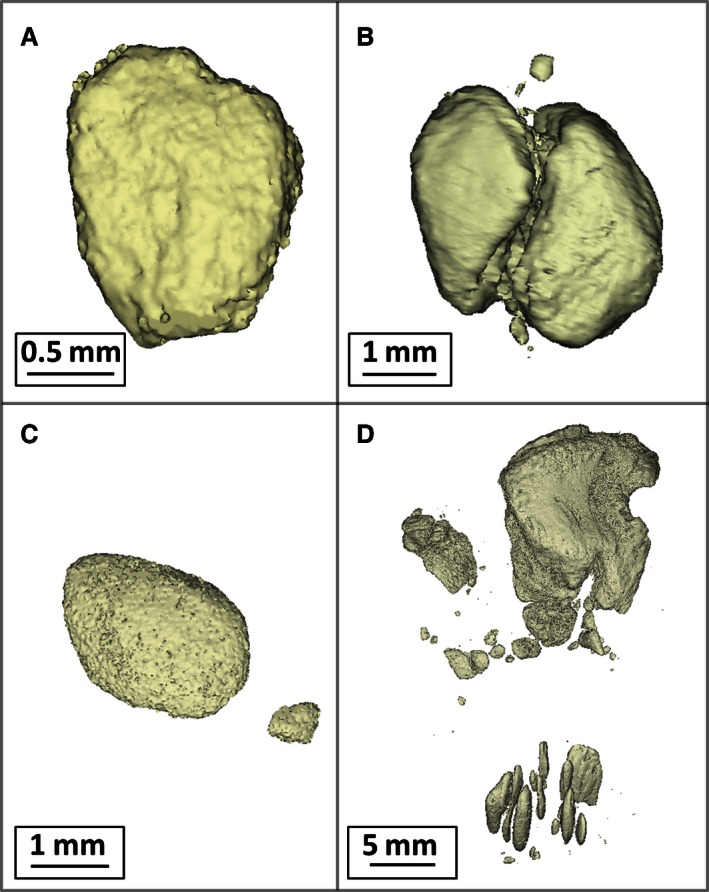
Morphology of the patella in XMT‐scanned squamates (viewing superficial surface, where top of image = proximal and bottom = distal). Generally the patellar mineralisation was flattened and ovoid in shape (e.g. (A) *Basiliscus plumifrons*). However in some scanned specimens, the patella appeared composed of multiple fusing parts similar to *Sphenodon* specimen ‘S1’ [(B) *Hydrosaurus pustulatus* with two main parts], or the patellar tendon contained multiple mineralised regions [(C) *Corucia zebrata* with two patellar mineralisations; (D) *Varanus ornatus* with multiple patellar mineralisations].

High‐resolution CT scans of lizard patellae show it generally comprised a single mineralised mass; 11 of 14 individuals/14 species showed a single mineralisation. This appears true of the radiographic images, too, although details are less clear due to lower image resolution and superimposition associated with this imaging modality. However, in three CT‐scanned lizards (*Corucia zebrata*,* Hydrosaurus pustulatus* and *Varanus ornatus*; detailed in Table 2), there were multiple or multipartite mineralisations within the patellar tendon (Fig. 5).

**Table 2:** Specimens that underwent scanning and histological examination in this study

Specimen	Patellar mineralisation?	Histological appearance
*Gekko gecko*	Yes (single)	Calcified hyaline cartilage
*Oplurus cuviers*	Yes (single)	Bone/appearance of calcified tendon with chondrocyte‐like cells
*Heloderma suspectum*	Yes (single)	Bone/appearance of calcified tendon with chondrocyte‐like cells
*Timon lepidus*	Yes (single)	Bone/appearance of calcified tendon with chondrocyte‐like cells
*Basiliscus plumifrons*	Yes (single)	Bone/calcified hyaline cartilage
*Corucia zebrata*	Yes (one main mineralisation with smaller one nearby)	Lamellar bone/appearance of calcified tendon with chondrocyte‐like cells
*Sceloporus serrifer*	Yes (single)	Appearance of calcified tendon with chondrocyte‐like cells
*Uromastyx sp*.	Yes (single)	Bone/appearance of calcified tendon with chondrocyte‐like cells/hyaline cartilage
*Hydrosaurus pustulatus*	Yes (multipartite with two main mineralised parts)	Appearance of calcified tendon with chondrocyte‐like cells
*Chamaeleo* sp. (cf. *C. chamaeleon*)	No	Region of very cellular, cartilage‐like tissue within typical vertebrate tendon
*Iguana iguana*	Yes (single)	Appearance of calcified tendon with chondrocyte‐like cells
*Tiliqua scincoides*	Yes (single)	Bone/fibrocartilage/appearance of calcified tendon/hyaline cartilage
*Chlamydosaurus kingii*	No	Some cartilage‐like tissue within typical vertebrate tendon
*Varanus ornatus*	Yes (multipartite with many mineralised parts)	Haversian and lamellar bone/appearance of calcified tendon with chondrocyte‐like cells/calcified hyaline cartilage
*Varanus* sp. (cf. *V. exanthematicus*)	Yes (single)	Appearance of calcified tendon with chondrocyte‐like cells
*Varanus komodoensis*	Yes (single)	N/a (histology not performed, but presume osseous from trabecular bone appearance of CT scan)
‘S1’ *Sphenodon punctatus*	Yes (multipartite with three main mineralised parts)	Appearance of calcified tendon with chondrocyte‐like cells
‘S15’ *Sphenodon punctatus*	No	Typical vertebrate tendon
BMNH1969.2204 *Sphenodon punctatus*	No	Typical vertebrate tendon

Histologically, some lizard patellae resembled the tissue micro‐structure of specimen ‘S1’, the sampled *Sphenodon*, with a tidemark demarcating the mineralised border, over which travelled continuous tendon fibres with chondrocyte‐like cells between them (Fig. 3C,D). Others, however, had a very different microscopic appearance, being composed of calcified hyaline cartilage (Fig. 3E), bone or various combinations of the above (e.g. bone, hyaline cartilage and/or calcified tendon with chondrocyte‐like cells; Fig. 3D,F).

**Figure 6:**
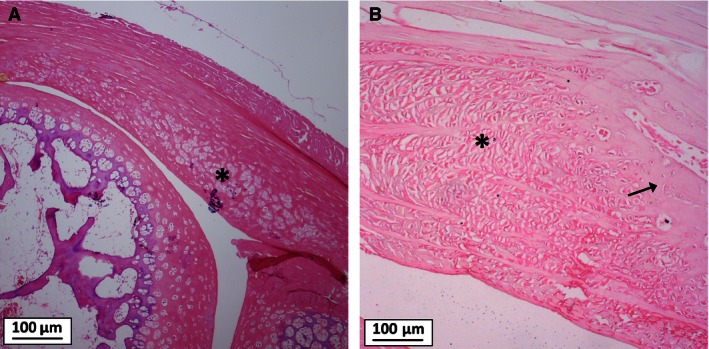
Histology of other regions of interest in the patellar tendon of squamates. (A) Patellar tendon of *Chamaeleo* sp. with an expanded region containing many chondrocyte‐like cells (asterisk) at the approximate location of the ossified patella in other squamates (the distal femur is visible in the bottom left of the image and a calcified lunula is in the bottom right). (B) A ‘suprapatellar’ region composed of cartilage‐like tissue was observed in some lizards such as *Tiliqua*, closely attached to the proximal pole of the ossified patella (arrow).

*Chamaeleo* sp. and *Chlamydosaurus kingii* both lacked ossified patellae in the individuals we studied, corroborated by available literature. However, in histological sections *Chamaeleo* sp. had a region within the patellar tendon containing many large cells, resembling chondrocytes, with tendon fibres running between them (Fig. 6A). *Chlamydosaurus kingii* also had a similar cellular region, albeit smaller. Some lizards (*Varanus* sp. and *Tiliqua scincoides*, both with osseous patellae) had similar regions within the patellar tendon more proximally, attached to the patella, but many other lizards did not (Fig. 6B).

### The patella in fossil Rhychocephalia and other Lepidosauromorpha

No clear evidence of patellar mineralisation was found in any of the fossil specimens examined in this study. Occasional mineralised structures were found in the general region of the knee, but non‐patellar explanations could not be excluded (e.g. displaced bone fragments, other small bones or scutes). No clear *in situ* patellar sesamoids were present in the study fossils or reported in literature on non‐squamate lepidosaurs, but as detailed in Supporting Information Data S1, there are numerous fossil squamates preserved with unambiguous patellae. For ancestral state reconstruction, fossil Rhynchocephalia were coded ‘?’ (unknown patellar state), and stem Lepidosauromorpha (e.g. Kuehneosauridae) were coded ‘0’ (ossified patella absent), considering our observations.

### Reconstruction of ancestral patellar state in Lepidosauria

**Figure 7:**
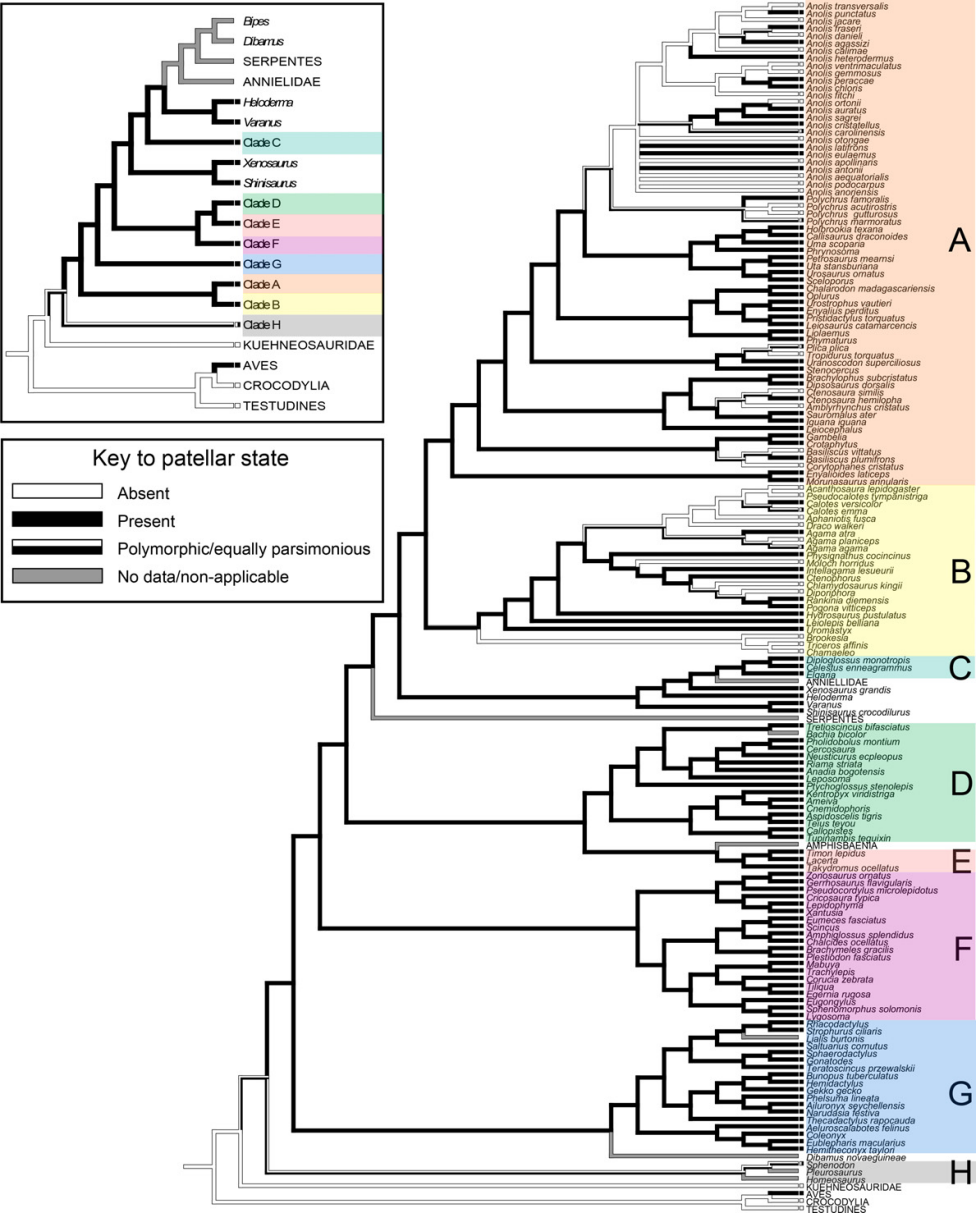
Parsimony ancestral state reconstruction over a composite lepidosaur tree built from Reeder et al. (2015) for main topology and Pyron et al. (2013) for genus branch order. Inset: simplified representation of the reconstruction over a morphologically derived tree (based on Gauthier et al. 2012) achieves a similar result basally (patella is ancestral for Squamata). Clade A = Dactyloidae + Polychrotidae + Phrynosomatidae + Opluridae + Leiosauridae + Liolaemidae + Tropiduridae + Iguanidae + Leiocephalidae + Crotaphytidae + Corytophanidae + Hoplocercidae; Clade B = Agamidae + Chamaeleonidae; Clade C = Anguidae; Clade D = Gymnophthalmidae + Teiidae; Clade E = Lacertidae; Clade F = Scincoidea; Clade G = Gekkota; Clade H = Rhynchocephalia.

Parsimony ancestral state reconstruction over our composite phylogeny supports the inference that an ossified patella is at least synapomorphic for the clade Squamata (Fig. 7), with later instances of loss among clades (e.g. Chamaeleonidae), species (e.g. *Chlamydosaurus kingii*) or individuals (apparently polymorphic taxa; e.g. *Polychrus marmoratus*).

The ancestral state for Lepidosauria as a whole depends heavily on the state assigned to members of Rhynchocephalia (and particularly *Sphenodon*). If coded as polymorphic within *Sphenodon* (state of ‘0/1’), the ancestral patellar state is reconstructed as equivocal for Lepidosauria. If *Sphenodon* is assigned state ‘1’ (mineralised patella present), a patella is reconstructed as ancestral for Lepidosauria, and likewise if the patella were to be coded ‘0’ (patella absent), a patella would be reconstructed as absent at the lepidosaurian root. These parsimony‐reconstructed ancestral states for Squamata and Lepidosauria remain the same when a morphology‐derived tree topology is used, such as one based on Gauthier et al. (2012) (Fig. 7, inset).

## Discussion

Our study has found the first evidence of a mineralised patellar sesamoid in several *Sphenodon* specimens, in contrast to previous literature that asserted that this genus lacks such a structure (e.g. Günther, 1867; Osawa, 1897; von Wettstein, 1931). Given its position within the patellar tendon over the distal femur and similarities to certain lizard patellae in morphology and composition, we conclude that this structure is potentially homologous to the patella in lizards, and certainly should be referred to as a patella even if it were convergently evolved. We attribute our finding of the patella in tuatara to the use of XMT scanning – an imaging modality with clear advantages over radiography and/or dissection for the detection of small skeletal elements – as well as to the infrequent presence of an ossified patella in seemingly skeletally mature tuatara (patellae were only present in four of 11 tuatara with complete epiphyseal fusion). Ossification sequence studies in lizards (Maisano, 2002a; Jerez et al. 2010) suggest that sesamoids such as the patella ossify relatively late compared with other skeletal elements (this is true of other species, though the patella itself is one of the earliest ossifying sesamoids; Vickaryous & Olson, 2007). Our squamate data circumstantially support this because we found no patellae in specimens with early epiphyseal ossification and more in specimens with greater degrees of epiphyseal fusion. Tuatara are very long‐lived and slow growing (Castanet et al. 1988), and so the apparent variability in patellar presence could be because the patella has yet to form in some individuals. Another possible explanation might be that the tuatara specimens sampled represent different populations (such as island groups) or lifestyles. An important and unfortunate limitation of our study is the lack of specimen history (age, provenance, etc.) which would be invaluable for distinguishing between these alternative explanations. A final possibility is that the patella is a polymorphic trait among tuatara. Sesamoids are known to exhibit variability between or even within individuals, e.g. the fabella (Vickaryous & Olson, 2007; Jerez et al. 2010). Although the patella is presumed monomorphic in squamates (as it is in mammals and to some extent birds), relatively few lepidosaurian individuals and species have been sampled to enable testing this.

An important aim of our study was to identify when and how the patella evolved in Lepidosauria, because an accurate reconstruction of phylogenetic history is the first step in understanding ‘why’ a trait has evolved. As might be expected, reconstruction of ancestral patellar state at the base of Lepidosauria depends heavily on patellar coding in Rhynchocephalia (*Sphenodon* and related taxa). This is problematic due to the overwhelming majority of rhynchocephalians being extinct and the additional difficulties in ascertaining patellar state in fossils. As in extant animals, the patella may appear to be absent because of its small size or late ossification. In fossils, there are further complications: the relative rarity of well‐preserved, articulated postcranial material; the fact that the patella may be less ossified (or only calcified) and less likely to be preserved; that it may be displaced more easily (being enveloped by soft tissue) and hard to identify if displaced (lacking a characteristic shape like some other short bones); finally, that it might be lost during fossil preparation, or conversely, remain unexposed in the matrix. The absence of evidence of the patella amongst fossil Rhynchocephalia (e.g. Renesto, 1995; Reynoso, 2000) does not necessarily equate to evidence of absence. Even within Squamata, for which we infer it is very likely to be ancestral (see below), the patella is seen only in certain exceptionally preserved fossil specimens (e.g. Evans et al. 2007; Conrad et al. 2011; Daza et al. 2013). Because of the uncertainty in the patellar state of its fossil relatives within Rhynchocephalia, coding choice in *Sphenodon* determines the lepidosaurian ancestral state in our reconstructions (see results). However, the presence of the patellar in *Sphenodon* at least, raises the possibility that presence of an ossified patella (in adult individuals) is synapomorphic for Lepidosauria as a whole (as ventured by Maisano, 2002a). This would place the origins of the ossified patella in a common ancestor as early as 250 mya (Jones et al. 2013).

Like Maisano (2002a), we find that the patella is present in many lizards (Fig. 7 and Supporting Information Data S1), and our ancestral state reconstructions support the hypothesis that it is a shared synapomorphy of squamates and was present in their common ancestor, around 200 mya (Hedges et al. 2015). However, within Squamata it seems to have been lost a number of times (among clades, e.g. Chamaeleonidae, or species, e.g. *Chlamydosaurus*; in addition to those taxa with highly reduced/absent hindlimbs) with some additional instances of seeming reversal (e.g. *Calotes* has a patella, but a patella is reconstructed as absent at its clade root of Draconinae; Fig. 7). As in the tuatara, but unlike in previous studies, we have found that the patella appears to be polymorphic in some squamates (e.g. *Polychrus marmoratus*). This would be different from what is known about the patella in birds and mammals, but not unusual for a sesamoid. Further careful study is needed to test whether the patella is truly polymorphic in these lizard taxa or whether our results might be due to other factors (e.g. very late ossification).

Our estimate of the phylogenetic history of the patella allows us to identify functional associations and begin making inferences regarding patellar evolution. A biomechanically adaptive hypothesis is generally cited (or implied) in explaining the presence or absence of the patella (Futuyma, 2015), but it has not been specifically evaluated in lizards or other reptiles. We asked: why is the patella present in lepidosaurs but not in other sprawling reptiles (e.g. crocodylians)? Our data alone cannot answer this question, but can begin testing pre‐existing hypotheses and generating new ones.

The presence of a patella in Squamata and Rhynchocephalia (and possibly the common ancestor of both) is consistent with the hypothesised link to secondary epiphyseal ossification centres or perhaps a general ‘ability to ossify’ various soft tissues (characters 33 and 34 in Gauthier et al. 1988). However, the origin of the patella in lepidosaurs is also closely associated with the evolution of specialised knee joint anatomy in this group, as described by Gauthier et al. (1988, character 27), with markedly asymmetrical femoral condyles and fibular contact with the lateral femur. Correspondingly, the only lizard clade that seems to have universally lost the patella (without evidence of re‐gain or polymorphism) is Chamaeleonidae. Chamaeleonidae are also the only (Recent) squamates noted to have symmetrical condyles (Rewcastle, 1980; Gauthier et al. 1988). The asymmetry of the condyles in most lizards and tuatara facilitates parasagittal knee extension despite their sprawling, non‐erect posture (Rewcastle, 1980). In mammals, the patella functionally increases the moment arm of the main knee extensor muscles (Haines, 1969; Alexander & Dimery, 1985; Fox et al. 2012). Therefore, we hypothesise that the presence of a patella would be more biomechanically advantageous in lepidosaurs with relatively planar knee movement and higher extensor muscle forces than in other Reptilia (e.g. crocodylians). More data on knee conformation and locomotion in reptiles would test this apparent correlation. Our observations on the lack of patella in Chamaeleonidae prompted this hypothesis, but we must mention that an observation by Pearson & Davin (1921) contradicts our data: they noted an ossified patella in *C. chamaeleon*. Other observations from this species – sesamoid at the proximal fibula, lack of lunulae and fabellae – also do not match our own. It is possible that the specimen was skeletally immature, and the ‘patella’ and ‘sesamoid’ were actually unfused epiphyseal ossifications. However, Chamaeleonidae are a large clade and there might be unnoticed diversity; more studies are needed.

Another aim of our study was better to characterise the morphology and composition of the patella in lepidosaurs. The bony patella is formed by endochondral ossification in birds and mammals, but we have found that this does not always appear to be the case in Lepidosauria. In some lizards there was evidence of a calcifying hyaline anlage, but in others there appeared to be direct mineralisation (i.e. calcification or ossification) of the tendon (which often contained chondrocyte‐like cells diffusely, in small clusters or rows). This tissue resembles fibrocartilage (like that noted in the quadriceps tendon by Clark & Stechschulte, 1998), and is also consistent with the ‘fibrovesicular tissue’ or ‘tendino‐vesicular tissue’ described by Haines (1940, 1969). Like Haines’ observations, our study specimens suggest that this tissue is sometimes replaced by bone, although with our static histological sampling we could not confirm that ossification was always the inevitable mature morphology. Composition of the patella was generally mixed (calcified hyaline cartilage, calcified tendon with chondrocyte‐like cells, and/or bone tissue; Table 2) which may suggest progression, but several seemingly mature lizards had no ossification. Haines also mentions a suprapatellar structure composed of ‘fibrovesicular’ tissue, which we observed infrequently in our sampled specimens (only *Varanus ornatus* and *Tiliqua scincoides*) attaching to the proximal pole of the ossified patella.

We have found that the patella in lizards and tuatara is occasionally multipartite, with parts sometimes connected as if fusing. Ossification from multiple centres is not unusual for sesamoids (Sarin & Carter, 2000; Hutchinson et al. 2011), and the patella in humans sometimes develops from multiple coalescing centres of ossification (Ogden, 1984; Dwek & Chung, 2008). Ossification studies that include the patella have been performed for a few other species (e.g. Hogg, 1980; Bland & Ashhurst, 1997) but as far as we are aware, none have noted multipartite patellae or multiple ossification centres. Sesamoids are highly sensitive to the mechanical environment of the limb (Sarin et al. 1999), and modelling studies suggest that ossification is initiated in regions of high tissue stress, explaining why sesamoids often have multiple centres of ossification (Sarin & Carter, 2000). Related to this is the idea that, evolutionarily, sesamoid bones may have initially formed as a phenotypic response (e.g. to a novel mechanical environment in the limb, such as one produced following changes in posture or locomotion), and later become ‘genetically assimilated’ (Sarin et al. 1999).

The similarities between the patella of *Sphenodon* and many squamates in position, morphology and histology support the hypothesis that the patella is a shared structure in lepidosaurs. It may be inherited from a common ancestor, or evolved through a similar developmental pathway (i.e. parallel evolution). When ‘lost’, the ossified patella seems to transition to a soft tissue ‘patelloid’ (e.g. the *Chamaeleo* sampled in this study, and perhaps also our *Chlamydosaurus*), similar to that of certain marsupials (Reese et al. 2001). A mineralised patella that alters joint mechanics may not provide a benefit in these species, but a soft tissue or fibrocartilage patelloid would continue to be an adaptation to resisting tendon shear (Benjamin et al. 1995). Fibrocartilage is routinely present in the regions where tendons are compressed, and may predispose the tendon to ossification (Benjamin et al. 1995). Although much more sampling is required in other lizards lacking the bony patella, our initial findings indicate a stepwise evolution from patella to patelloid, and raise the possibility of the reverse – a similar soft tissue precursor facilitating evolution of the osseous patella in the lepidosaurian ancestor.

To summarise, we have found intriguing new evidence for the patella as a synapomorphy of Lepidosauria, which would represent the earliest instance of patellar evolution at ~ 250 mya [vs. ~ 70 mya for birds (Regnault et al. 2014) and ~ 175 mya in mammals (Samuels et al. manuscript in preparation)]. However, our conclusions are somewhat limited by lack of specimen history (e.g. tuatara ages, provenance) and inconclusive fossil evidence. It is difficult to prove the absence of a patella in fossils but we hope careful examination with newer technologies (e.g. UV light photography, fossil XMT) and an awareness of past pitfalls (e.g. over‐preparation) will result in more data to test whether the patella is ancestral for lepidosaurs. The surprising amount of patellar variation and polymorphism observed in this study suggests that further sampling may uncover additional diversity and reveal more subtle patterns of form and function. We speculate that sesamoids such as the patella may have initially formed as a by‐product (or spandrel) of other physiological processes (e.g. tendon metaplasia, general ossification ability, changes in limb mechanics), then later ‘exapted’ (co‐opted as fixed adaptations) due to biomechanical benefit(s) provided to the animal. We aim to investigate the nature of these benefits in future work through experimentation and modelling.

## Author contributions

The study was conceived by JRH. Data were collected by SR and MEHJ, and interpreted by SR, MEHJ, JRH and AAP. The manuscript and figures were produced by SR, with significant input from JRH, MEHJ and AAP.

## Supporting information


**Table S1.** *Sphenodon* and lizard specimens with their imaging parameters.
**Table S2.** Fossil specimens examined in this study.

**Data S1.** An excel (.xlsl) file with the patellar character data for squamate taxa, used for ancestral state reconstruction in this paper.
