# Supplementary material for: Anatomy, morphology and evolution of the patella in squamate lizards and tuatara (Sphenodon punctatus)
Source: J Anat. 2016 Jan 6;228(5):864–76. doi: 10.1111/joa.12435 (PMC4831346; doi:10.1111/joa.12435)
Supplement: Supplementary file 1 — Table S1. Sphenodon and lizard specimens with their imaging parameters. Table S2. Fossil specimens examined in this study. [file JOA-228-864-s001.docx]

**Supplementary Material**

Supplementary Data file 1: An excel (.xlsl) file with the patellar character data for squamate taxa, used for ancestral state reconstruction in this paper.

| Specimen | Imaging machine and parameters |
| --- | --- |
| *Sphenodon punctatus* (no official research number, assigned “S1” here) from Marc Jones’ personal collection held at Evans Lab, University College London (UCL) | XT H 225 ST computed tomography system (Nikon Metrology, Brighton MI USA), CT settings: 145kV, 280µA, 1415ms, voxel size 0.093mm, 1908 slices  Skyscan 1172 (Bruker microCT, Kontich Belgium), CT settings: 49kV, 200µA, 2100ms, voxel size 5.00µm, 2010 slices |
| *Sphenodon punctatus*, specimen numbers R.2595, R.2596, R.2598, R.2602, R.2603, R.2604, R.2605, R.2607, R.2608, R.2609, R.2615, and R.2616 belonging to University Museum of Zoology Cambridge | XT H 225 ST computed tomography system (Nikon Metrology, Brighton MI USA), CT settings:  R.2595 - 94kV, 466µA, 1000ms, voxel size 0.098mm, 1001 slices  R.2596 - 94kV, 466µA, 1000ms, voxel size 0.089mm, 1001 slices  R.2598 - 91kV, 466µA, 1000ms, voxel size 0.101mm, 1001 slices  R.2602 - 76kV, 790µA, 1000ms, voxel size 0.120mm, 1001 slices  R.2603 - 71kV, 665µA, 1000ms, voxel size 0.084mm, 1001 slices  R.2604 - 94kV, 466µA, 1000ms, voxel size 0.093mm, 1001 slices  R.2605 - 94kV, 466µA, 1000ms, voxel size 0.094mm, 1001 slices  R.2607 - 145kV, 295µA, 1000ms, voxel size 0.125mm, 1001 slices  R.2608 - 69kV, 635µA, 1000ms, voxel size 0.114mm, 1001 slices  R.2609 - 150kV, 270µA, 1000ms, voxel size 0.097mm, 1001 slices  R.2615 - 115kV, 245µA, 1000ms, voxel size 0.125mm, 1001 slices  R.2616 - 110kV, 235µA, 1000ms, voxel size 0.125mm, 1001 slices |
| *Sphenodon punctatus* (no official research number, assigned “S15” here) belonging to Queen Mary University of London | XT H 225 ST computed tomography system (Nikon Metrology, Brighton MI USA), CT settings: 70kV, 465µA, 1000ms, voxel size 0.092mm, 1081 slices |
| *Sphenodon punctatus* (no official research number, assigned “S16” here) belonging to University of Helsinki | XT H 225 ST computed tomography system (Nikon Metrology, Brighton MI USA), CT settings: 95kV, 516µA, 1000ms, voxel size 0.108mm, 1081 slices |
| *Sphenodon punctatus*, specimen numbers NH.3.116 and NH.84.19 belonging to Horniman Museum London | XT H 225 ST computed tomography system (Nikon Metrology, Brighton MI USA), CT settings:  NH.3.116 - 75kV, 336µA, 1000ms, voxel size 0.110mm, 1081 slices  NH.84.19 - 75kV, 336µA, 1000ms, voxel size 0.089mm, 1081 slices |
| *Sphenodon punctatus*, specimen numbers BMNH1969.2204 and BMNH1935.12.6.1 belonging to Natural History Museum UK | XT H 225 ST computed tomography system (Nikon Metrology, Brighton MI USA), CT settings:  BMNH1969.2204 - 75kV, 360µA, 1000ms, voxel size 0.087mm, 1081 slices  BMNH1935.12.6.1 - 75kV, 360µA, 1000ms, voxel size 0.087mm, 1081 slices |
| *Gekko gecko* (no official research ID) belonging to Evans Lab UCL | Skyscan 1172 (Bruker microCT, Kontich Belgium), CT settings: 49kV, 200µA, 1600ms, voxel size 9.99µm, 1222 slices |
| *Oplurus cuviers* research ID ZR/193/11 belonging to Evans Lab UCL | Skyscan 1172 (Bruker microCT, Kontich Belgium), CT settings: 49kV, 200µA, 1600ms, voxel size 9.99µm, 1222 slices |
| *Heloderma suspectum* research ID ZR-101-10 belonging to Evans Lab UCL | Skyscan 1172 (Bruker microCT, Kontich Belgium), CT settings: 49kV, 200µA, 1600ms, voxel size 9.99µm, 1070 slices |
| *Timon lepidus* research ID ZR 137/09 belonging to Evans Lab UCL | Skyscan 1172 (Bruker microCT, Kontich Belgium), CT settings: 49kV, 200µA, 1600ms, voxel size 9.99µm, 1222 slices |
| *Basiliscus plumifrons* research ID ZR/519/09 belonging to Evans Lab UCL | Skyscan 1172 (Bruker microCT, Kontich Belgium), CT settings: 49kV, 200µA, 1600ms, voxel size 9.99µm, 1222 slices |
| *Corucia zebrata* research ID ZR/935/10 belonging to Evans Lab UCL | Skyscan 1172 (Bruker microCT, Kontich Belgium), CT settings: 49kV, 200µA, 1600ms, voxel size 9.99µm, 1298 slices |
| *Sceloporus serrifer* research ID ZR/058/10 belonging to Evans Lab UCL | Skyscan 1172 (Bruker microCT, Kontich Belgium), CT settings: 49kV, 200µA, 1600ms, voxel size 9.99µm, 1222 slices |
| *Uromastyx sp.* research ID ZR/190/10 belonging to Evans Lab UCL | Skyscan 1172 (Bruker microCT, Kontich Belgium), CT settings: 49kV, 200µA, 1600ms, voxel size 9.99µm, 1222 slices |
| *Hydrosaurus pustulatus* research ID ZR/922/10 belonging to Evans Lab UCL | Skyscan 1172 (Bruker microCT, Kontich Belgium), CT settings: 49kV, 200µA, 1600ms, voxel size 9.99µm, 1222 slices |
| *Chamaeleo* sp. (cf. *C. chamaeleon*) (no official research ID) belonging to Royal Veterinary College, UK | Skyscan 1172 (Bruker microCT, Kontich Belgium), CT settings: 49kV, 200µA, 1600ms, voxel size 9.99µm, 1033 slices |
| *Iguana iguana* no official research ID) belonging to Royal Veterinary College, UK | XT H 225 ST computed tomography system (Nikon Metrology, Brighton MI USA), CT settings: 80kV, 260µA, 1000ms, voxel size 0.087mm, 1081 slices  Skyscan 1172 (Bruker microCT, Kontich Belgium), CT settings: 49kV, 200µA, 2100ms, voxel size 5.00µm, 2908 slices |
| *Tiliqua scincoides* (no official research ID) belonging to Royal Veterinary College, UK | Skyscan 1172 (Bruker microCT, Kontich Belgium), CT settings: 49kV, 200µA, 3500ms, voxel size 13.5µm, 3034 slices |
| *Chlamydosaurus kingii* (no official research ID) belonging to Royal Veterinary College, UK | Skyscan 1172 (Bruker microCT, Kontich Belgium), CT settings: 49kV, 200µA, 3500ms, voxel size 13.5µm, 1955 slices |
| *Varanus ornatus* (no official research ID) belonging to Royal Veterinary College, UK | Skyscan 1172 (Bruker microCT, Kontich Belgium), CT settings: 59kV, 167µA, 4500ms, voxel size 13.5µm, 3375 slices |
| *Varanus* sp. (cf. *V. exanthematicus*) (no official research ID) belonging to Royal Veterinary College, UK | XT H 225 ST computed tomography system (Nikon Metrology, Brighton MI USA), CT settings: 75kV, 336µA, 1000ms, voxel size 0.089mm, 1081 slices  Skyscan 1172 (Bruker microCT, Kontich Belgium), CT settings: 49kV, 200µA, 2100ms, voxel size 5.00µm, 2076 slices |
| *Varanus komodoensis* belonging to Zoological Society London | Lightspeed Pro 16 CT system (GE Medical, UK), CT settings: 120kV, 100mA, slice thickness 0.625mm, 509 slices |

Supplementary table 1: *Sphenodon* and lizard specimens with their imaging parameters

| Fossil taxa | Specimen numbers |
| --- | --- |
| *Homeosaurus* (Rhynchocephalia) | MfN:  MB.R.1011.1, MB.R.1013, MB.R.3799, MB.R.1014, MB.R.1007.2, MB.R.893.2  SMNS: 81200, 81201, 81203, 10888  NHMUK: R.2741 |
| *Kallimodon* (Rhynchocephalia) | MfN:  MB.R.1009.2, MB.R.1008.2, MB.R.1012.1  NHMUK: R.1693, R.2741 |
| *Sapheosaurus* (Rhynchocephalia) | MfN: MB.R.1949  NHMUK: R.2692 |
| *Kuehneosaurus* (stem lepidosaur) | NHMUK: R.6111, R.6112, R.6114, R.6115, R.6113, R.8172 |
| *Clevosaurus* (Rhynchocephalia) | UMZC: T1271 |
| *Paramacellodus owenii* (Squamata) | NHMUK: R.8209 |
| *Brachyrhinodon taylori* (Rhynchocephalia) | NHMUK: R.4776, R.4777 |
| *Saurosternon bainii* (Neodiapsida) | NHMUK: R.1234 |
| *Aphelosaurus lutevensis* (Araeoscelidia) | NHMUK: R.49658 |
| *Galesphyrus capensis* (Neodiapsida) | NHMUK: R.4084 |
| *Tanystropheidae incertaesedis* (Protosauria) | SMNS: no ID |
| *Askeptosaurus ilalicus* (Thalattosauria) | SMNS: 81794 |
| *Bavarisaurus* (Squamata) | SMNS: 81785 |
| *Iguania* (Squamata) | SMNS: 81202 |
| *Palaeopleurosaurus posidoniae* (Rhynchocephalia) | SMNS: 50721, 50722, 81774 |
| *Pleurosaurus* (Rhynchocephalia) | MfN: MB.R.1948, MB.R.1949  SMNS: 56604, 55058, 58020, 81844  NHMUK: R.3934 |
| *Protorosaurus speneri* (Protosauria) | MfN: MB.R.2169, MB.R.2168.1-2  SMNS: 56421, 59790 |
| *Stereosternum tumidum* (Mesosauria) | R142c (MfN) |
| *Eichstättisaurus schroederi* (Squamata) | MB.R.2035 (MfN) |

Supplementary table 2: Fossil specimens examined in this study. Abbreviations: MfN = [Museum für Naturkunde](http://www.naturkundemuseum-berlin.de/en/) Berlin, SMNS = Staatliches Museum für Naturkunde Stuttgart, NMHUK = Natural History Museum London, UMZC = University Museum of Zoology Cambridge.
